# 4500 Providing a System for Practical Monitoring Training for Clinical Trials within Academic Institutions

**DOI:** 10.1017/cts.2020.348

**Published:** 2020-07-29

**Authors:** Advaita Chandramohan, Sukhmani Kaur, Eunjoo Pacifici

**Affiliations:** 1 University of Southern California

## Abstract

OBJECTIVES/GOALS: The goal was to understand the effectiveness of a novel clinical trial educational module and a corresponding initiative designed and disseminated by the Southern California Clinical and Translational Science Institute (SC-CTSI) to increase the quality of clinical trials conducted in academia. METHODS/STUDY POPULATION: The CRCs (Clinical Research Coordinators) for the initiative are asked to complete the online training. Possible study protocols are picked to be monitored by the CRCs. The monitor is instructed to study the protocol extensively and prepare for their monitoring visit. The trained monitor from the initiative then reaches out to the CRC of the study that is to be monitored and carries out the monitoring visit. Afterwards, the monitor sends initiative personnel the monitoring report, which is evaluated to see if the monitor checked everything they should have during the visit. The PI of the study is contacted with highlights from the monitoring report and improvements that they can make. RESULTS/ANTICIPATED RESULTS: The first study monitored was a site of a large NIH-sponsored study where the consent forms were signed electronically. It was found that the monitor could not access the consent forms. Therefore, the monitor could not do source data verification. The PI of the study said that they would be raising this issue with the NIH. During the monitoring visit of the second study chosen for the initiative, patient binders were specifically examined for informed consent and source documentation completeness. The charts of patients were also reviewed. The only deviation found was a missing signature in the Investigator Site File. For the last two studies, data will be reported. DISCUSSION/SIGNIFICANCE OF IMPACT: Monitors were not only able to monitor efficiently, but also able to point out deficiencies in the monitoring practices of large studies. This model could be expanded to other academic institutions to establish quality management systems to ensure data integrity and subject protection.

